# Genetic polymorphisms in *TLR3*, *IL10* and *CD209* influence the risk of BK polyomavirus infection after kidney transplantation

**DOI:** 10.1038/s41598-022-15406-0

**Published:** 2022-07-05

**Authors:** Natalia Redondo, Isabel Rodríguez-Goncer, Patricia Parra, Francisco López-Medrano, Esther González, Ana Hernández, Hernando Trujillo, Tamara Ruiz-Merlo, Rafael San Juan, María Dolores Folgueira, Amado Andrés, José María Aguado, Mario Fernández-Ruiz

**Affiliations:** 1 Unit of Infectious Diseases, Hospital Universitario “12 de Octubre”, Instituto de Investigación Sanitaria Hospital “12 de Octubre” (imas12), Centro de Actividades Ambulatorias, 6ª planta, Bloque D. Avda. de Córdoba, s/n, 28041 Madrid, Spain; 2 Centro de Investigación Biomédica en Red (CIBER) en Enfermedades Infecciosas, Madrid, Spain; 3 Department of Medicine, School of Medicine, Universidad Complutense, Madrid, Spain; 4 Department of Nephrology, Hospital Universitario “12 de Octubre”, Instituto de Investigación Sanitaria Hospital “12 de Octubre” (imas12), Madrid, Spain; 5 Department of Microbiology, Hospital Universitario “12 de Octubre”, Instituto de Investigación Sanitaria Hospital “12 de Octubre” (imas12), Madrid, Spain

**Keywords:** Genetics research, Risk factors, Genetic predisposition to disease, Infection

## Abstract

Genetic determinants of BK polyomavirus infection after kidney transplantation remain poorly investigated. We assessed the potential impact of 13 different single nucleotide polymorphisms within genes mainly involved in innate immune responses on the risk of BKPyV viremia in 204 KT recipients. After a median follow-up of 1121.5 days, the cumulative incidence of any-level BKPyV viremia was 24.5% (50/204). There was a significant association between the minor T allele of *TLR3* (rs3775291) SNP and the development of BKPyV viremia (adjusted hazard ratio [aHR]: 2.16; 95% confidence interval [CI]: 1.08–4.30; *P* value = 0.029), whereas the minor G allele of *CD209* (rs4804803) SNP exerted a protective role (aHR: 0.54; 95% CI: 0.29–1.00; *P* value = 0.050). A higher incidence of BKPyV viremia was also observed for the minor G allele of *IL10* (rs1800872) SNP, although the absence of BKPyV events among homozygotes for the reference allele prevented multivariable analysis. The BKPyV viremia-free survival rate decreased with the increasing number of unfavorable genotypes (100% [no unfavorable genotypes], 85.4% [1 genotype], 70.9% [2 genotypes], 52.5% [3 genotypes]; *P* value = 0.008). In conclusion, SNPs in *TLR3*, *CD209* and *IL10* genes play a role in modulating the susceptibility to any-level BKPyV viremia among KT recipients.

## Introduction

The innate immunity plays important roles in mounting protection against viral infection, which are mediated by several classes of pathogen recognition receptors (PRRs)^1^. The families described so far include toll-like receptors (TLRs), retinoic acid-inducible gene I (RIG-I)-like receptors, nucleotide-binding oligomerization domain-like receptors, and C-type lectin receptors (CLRs)^2,3^. Most of them are able to recognize the presence of conserved molecular motifs on pathogenic pathogens (such as double-stranded viral RNA [dsRNA]), known as pathogen-associated molecular patterns (PAMPs)^4^. Upon recognition of PAMPs by PRRs, signal transduction pathways are quickly triggered to converge on nuclear transcription factors and activate the innate machinery as the first line of host defense^5^.

It is well established that the genetic diversity revealed by single-nucleotide polymorphisms (SNP) within genes coding for immune mediators—such as the mentioned PRRs or interleukins (ILs)—modulates the individual susceptibility to viral pathogens. Since the immunosuppressive therapies used in transplant recipients are mainly targeted at the adaptive arm, the relative contribution of the innate immunity becomes more obvious than in non-transplant patients. Indeed, SNPs located in different members of the TLR family have been shown to impact on the risk of post-transplant cytomegalovirus (CMV) infection^3,6–8^, and similar associations have been also described for CLRs such as mannose-binding lectin^9,10^ or dendritic-cell specific ICAM 3-grabbing nonintegrin (DC-SIGN or CD209)^8^.

BK polyomavirus (BKPyV) is a double-stranded DNA virus belonging to the *Polyomaviridae* family^11^. Primary infection occurs during early childhood and is usually asymptomatic, whereas seroprevalence rates exceed 90% in the adult population^12,13^. The reactivation of latent BKPyV infection may result in the development of BKPyV-associated nephropathy (BKPyVAN), a major cause of graft loss after kidney transplantation (KT)^14,15^. Various patient-, procedure- and immunosuppression-related factors—including deceased donor, longer cold ischemia time, ABO incompatibly or tacrolimus-containing regimens, among others—have been shown to increase the risk of BKPyV infection^16,17^. However, the predictive accuracy of current risk stratification strategies is still suboptimal and obliges physicians to closely monitor for BKPyV viruria or viremia in an attempt to detect reactivation as early as possible.

The cellular receptor for BKPyV is an α(2,3)-linked sialic acid that facilitates virus entry through a caveolae-mediated endocytosis process^11,18–20^. Once in the cytoplasm, the virus migrates to the nucleus where replication and transcription take place^11,21^. BKPyV establishes latency in the urothelial cells and occasionally reactivates in healthy individuals, resulting in transient viruria^11,22^. Most of the existing research on the host immunity against BKPyV has been focused on adaptive T-cell responses^23–25^ or on the role of dendritic cells (DCs)^26^, HLA alleles^27^ or defensins^11,28^. A few studies have investigated the effect of some SNPs in genes encoding interferon (IFN)-γ^29^ or Fc gamma receptors^30^, with conflicting results. Nevertheless, little is still known on how the innate immunity interacts with BKPyV, with some evidence pointing to the role of TLR3 in the immune cascade triggered by the virus^18^.

To gain insight into the effect of innate immune responses on the risk of BKPyV viremia in KT recipients, we have analyzed 13 different SNPs within a set of genes coding for molecules mainly involved in viral sensing (Table 1). The rationale for the choice of these SNPs is that some of them have been associated with the susceptibility to other viral infections in the transplant setting, as recently reviewed by our group^31^. In detail, the *TLR2* (rs5743708) SNP has been associated with an increased incidence of CMV infection and disease after liver transplantation*.* It has been also shown the impact of *TLR9 (*rs5743836) SNP on the occurrence of CMV infection in seropositive KT recipients. Regarding genetic polymorphisms located in *CD209,* rs735240 acts as a risk factor among CMV-seropositive KT recipients not receiving antiviral prophylaxis, whereas the rs4804803 SNP has been linked with the susceptibility to hepatitis B virus (HBV) or tick-borne encephalitis (TBE) virus, among others. The effect of *IFNL3* (rs12979860) SNP has been previously investigated in terms of BKPyV viremia and BKPyVAN, as well as CMV infection^31^. In addition we considered SNPs for which some prior evidence suggests an involvement in BKPyV infection or BKPyVAN, such as *TLR3* and *TNF*^18,32^. Finally, we included polymorphisms in the *CTLA4* gene, which codes for a costimulatory receptor crucial in immune homeostasis^33^.

**Table 1:** Description of the selected candidate SNPs for the present study.

Gene	Encoded protein	Biological function	SNP ID number	Nucleotide substitution (reference allele/alternative allele)	Global allele frequency^a^
CTLA4	Cytotoxic T-lymphocyte antigen 4 (CTLA-4/CD152)	T-cell co-inhibitory receptor	rs5742909	C/T	C = 0.91755 T = 0.08245
			rs231775	A/G	A = 0.628256 G = 0.371744
TLR2	Toll-like receptor 2: membrane-bound pattern recognition receptor	Pathogen recognition of large variety of microbial ligands and activation of innate immunity	rs5743708	G/A	G = 0.97373 A = 0.02627
TLR3	Toll-like receptor 3: endosomal pattern recognition receptor	Endocytic pathogen recognition receptor of single and double-stranded RNA	rs3775291	C/T	C = 0.716526 T = 0.283474
TLR9	Toll-like receptor 9: endosomal pattern recognition receptor	Recognition of unmethylated CpG motif-containing DNA	rs5743836	A/G	A = 0.80444 G = 0.19556
			rs352139	T/C	T = 0.458978 C = 0.541022
CD209	Dendritic cell-specific ICAM 3-grabbing nonintegrin (DC-SIGN/CD209): endosomal C-type lectin receptor	Recognition of carbohydrates present in viruses, bacteria, fungi and parasites and DAMPs in damaged host T-cells	rs735240	G/A	G = 0.57414 A = 0.42586
			rs4804803	A/G	A = 0.786719 G = 0.213281
IFNL3	Interferon-λ3 (IL28B), type III interferon: soluble immune mediator	Antiviral cytokine	rs12979860	C/T	C = 0.672446 T = 0.327554
			rs8099917	T/G	T = 0.808472 G = 0.191528
TNF	Tumor necrosis factor	Pro-inflammatory cytokine	rs1800629	G/A	G = 0.847933 A = 0.152067
IL10	Interleukin-10: human cytokine	Anti-inflammatory cytokine	rs1800872	T/G	T = 0.29385 G = 0.70615
			rs1878672	G/C	G = 0.68890 C = 0.31110

*DAMP* damage-associated molecular pattern; *SNP* single-nucleotide polymorphism.

^a^Obtained from ALFA Allele Frequency (available at: https://www.ncbi.nlm.nih.gov/snp/).

## Results

### Study population and incidence of BKPyV viremia

Overall we included 204 KT recipients, whose demographics and clinical characteristics are detailed in Table 2. The median follow-up period was 1121.5 days (interquartile range [IQR]: 955–1297), and all but 6 patients (97.1%) completed the 12-month follow-up with a functioning graft. Eleven patients (5.4%) died at a median of 963 days (IQR: 626–1312) from transplantation, whereas 8 patients (3.9%) experienced graft-loss (none of them attributable to BKPyV infection). One- and two-year death-censored graft survival rates were 98.5% and 95.9%, respectively.

**Table 2:** Demographics and clinical characteristics of the study cohort (n = 204).

Variable	
Age, years [mean ± SD]	54.6 ± 15.7
Gender (male) [n (%)]	146 (71.6)
Body mass index, Kg/m^2^ [mean ± SD]^a^	25.9 ± 9.5
**Ethnicity [n (%)]**	
Caucasian	177 (86.8)
Hispanic	17 (8.3)
African	6 (2.9)
Asian	4 (2.0)
Current or prior smoking history [n (%)]	81 (39.9)
**Pre-transplant chronic co-morbidities [n (%)]**	
Hypertension	175 (85.8)
Diabetes mellitus	58 (28.4)
Chronic lung disease	27 (13.2)
Coronary heart disease	21 (10.3)
Other chronic heart disease	35 (17.2)
Peripheral arterial disease	21 (10.3)
Cerebrovascular disease	17 (8.3)
Previous solid organ transplantation [n (%)]	28 (13.7)
**Underlying end-stage renal disease [n (%)]**	
Diabetic nephropathy	35 (17.2)
Polycystic kidney disease	24 (11.8)
Glomerulonephritis	55 (27.0)
Nephroangiosclerosis	18 (8.8)
Congenital nephropathy	8 (3.9)
Reflux nephropathy	7 (3.4)
NSAID-associated nephropathy	3 (1.5)
Amiloidosis	3 (1.5)
Unknown	25 (12.3)
Other	26 (12.7)
**CMV serostatus [n (%)]**	
D+/R+	148 (72.5)
D+/R−	23 (11.3)
D−/R+	22 (10.8)
D−/R−	7 (3.4)
D unknown/R +	4 (2.0)
Positive HCV serostatus [n (%)]^b^	15 (7.4)
Positive HIV serostatus [n (%)]^c^	2 (1.0)
Pre-transplant renal replacement therapy [n (%)]	180 (88.2)
Hemodialysis	148/180 (82.2)
Continuous ambulatory peritoneal dialysis	32/180 (17.8)
Time on dialysis, months [median (IQR)]	17.2 (8.9–35.4)
Age of donor, years [mean ± SD]	53.8 ± 15.5
Gender of donor (male) [n (%)]	109 (53.4)
**Type of donor [n (%)]**	
DBD donor	128 (62.7)
DCD donor	46 (22.6)
Living donor	29 (14.2)
Cold ischemia time, hours [median (IQR)]	18.0 (10.1–23.0)
Number of HLA mismatches [median (IQR)]	4 (3–5)
**Induction therapy [n (%)]**	
ATG	94 (46.1)
Basiliximab	83 (46.7)
None	27 (13.2)
**Primary immunosuppression regimen [n (%)]**	
Prednisone, tacrolimus and MMF/MPS	196 (96.1)
Prednisone, tacrolimus and azathioprine	8 (3.9)
Conversion to mTOR inhibitor during follow-up [n (%)]	19 (9.3)
Time to conversion, days [median (IQR)]	232 (118–321)
Anti-CMV prophylaxis [n (%)]	113 (55.4)
Duration of prophylaxis, days [median (IQR)]	103 (91–147)
**Post-transplant complications [n (%)]**	
Delayed graft function	99 (48.5)
Number of dialysis sessions [median (IQR)]	2 (1–3)
NODAT	24 (11.8)
CMV infection [n (%)]	114 (55.9)
CMV disease [n (%)]	22 (10.8)
Any-level BKPyV viremia [n (%)]	50 (24.5)
Renal artery stenosis	40 (19.6)
Acute graft rejection	25 (12.3)
Time from transplantation to the first episode, days [median (IQR]	134 (28.5–291.5)
T-cell-mediated acute rejection	16 (7.8)
Borderline T-cell-mediated rejection	8 (3.9)
Antibody-mediated acute rejection	5 (2.5)

*ATG* antithymocyte globulin; *BKPyV* BK polyomavirus; *CMV* cytomegalovirus; *D* donor; *DBD* donation after brain death; *DCD* donation after circulatory death; *HCV* hepatitis C virus; *HIV* human immunodeficiency virus; *HLA* human leukocyte antigen; *IQR* interquartile range; *MMF/MPS* mycophenolate mofetil/mycophenolate sodium; *mTOR* mammalian target of rapamycin; *NSAID* nonsteroidal anti-inflammatory drug; *R* recipient; *SD* standard deviation; *NODAT* new-onset diabetes after transplantation.

^a^Data on body mass index of the recipient was not available for 17 patients.

^b^Data on the HCV serostatus was not available for 5 patients.

^c^Data on the HIV serostatus was not available for 2 patients.

The median number of individual monitoring points for BKPyV viremia per patient during the first post-transplant year was 8 (IQR: 5–10). The presence of any-level BKPyV viremia (study outcome) was documented at any point during the follow-up period in 50 patients (24.5%) that developed 61 separate episodes, yielding 12- and 24-month cumulative incidences of 17.6% (36/204) and 21.6% (44/204), respectively (Fig. S1). The median interval between transplantation and the first documented episode of viremia was 184 days (IQR: 82.3–390), whereas the mean tacrolimus trough level at that time was 7.7 ± 2.6 ng/mL. Peak median viremia was 2.9 log_10_ copies/mL (IQR: 2.5–3.6). Recurrent viremia was observed in 5 patients (2.5%) whose peak median viremia was 4.01 log_10_ copies/mL (IQR: 3.9–5.1). Eight out of 50 patients (3.9%) had high-level DNAemia (> 4.0 log_10_ copies/mL), and three of them fulfilled the diagnostic criteria of presumptive BKPyVAN, which resulted in a cumulative incidence of 1.5%. Two out of eight patients (25.0%) with DNAemia > 4.0 log_10_ copies/mL underwent renal biopsy, and none of them had histological findings of BKPyVAN or positive immunohistochemical staining for SV40. In addition, there were no cases of BKPyV-associated hemorrhagic cystitis either.

Regarding graft outcomes, there were no differences in estimated glomerular filtration rates (eGFR) at the time of diagnosis and end of follow-up in the overall group of patients experiencing any BKPyV viremia (45.4 ± 18.4 vs. 43.9 ± 20.0 mL/min; *P* value for repeated measures = 0.589). Nevertheless, eGFR significantly decreased between both points in the subgroup of recipients with high-level DNAemia > 4.0 log_10_ copies/mL (59.6 ± 26.1 vs. 49.6 ± 24.2 mL/min; *P* value repeated measures = 0.031). In addition, eGFR at the end of follow-up was lower among patients that experienced any-level BKPyV DNAemia as compared to those that remained free from this event (53.1 ± 19.8 mL/min; *P* value = 0.024).

### Effect of SNPs on the cumulative incidence of BKPyV viremia

The 13 SNPs subjected to analysis are described in Table 1. All the samples were successfully genotyped. Only three SNPs—*CTLA4* (rs5742909), *TLR9* (rs5743836) and *IFNL3* (rs12979860)—deviated from the Hardy–Weinberg equilibrium (Table S1). The genotype frequency distribution of each SNP is detailed in Table S1.

Firstly, we performed an exploratory analysis to evaluate the correlation between the different genotypes of each SNP and the cumulative incidence of BKPyV viremia. We found a significant association between this event and the *IL10* (rs1800872) SNP. In detail, carriers of the minor G allele in heterozygosis (TG) or homozygosis (GG) exhibited a higher rate of BKPyV viremia as compared to homozygotes for the major allele (TT) (*P* value = 0.033). There was also a trend for the *TLR3* (rs3775291) SNP suggesting that patients with homozygous state of the minor T allele (TT) would show an increased risk of BKPyV viremia as compared to CC or CT carriers (*P* value = 0.078) (Table 3).

**Table 3:** Comparison of the cumulative incidence of BKPyV viremia according to different genotypes in candidate SNPs.

Gene (SNP database ID number)	Genotype	BKPyV viremia, n (%)		*P* value^a^
		No (N = 154)	Yes (N = 50)	
CTLA4 (rs5742909)	CC	127 (82.5)	40 (80.0)	0.712
	CT	24 (15.6)	8 (16.0)	
	TT	3 (1.9)	2 (4.0)	
CTLA4 (rs231775)	AA	77 (50.0)	27 (54.0)	0.882
	AG	61 (39.6)	18 (36.0)	
	GG	16 (10.4)	5 (10.0)	
TLR2 (rs5743708)	GG	153 (99.4)	50 (100.0)	0.568
	GA	1 (0.6)	0 (0.0)	
	AA	N/A	N/A	
TLR3 (rs3775291)	CC	79 (51.3)	22 (44.0)	0.078
	CT	60 (39.0)	17 (34.0)	
	TT	15 (9.7)	11 (22.0)	
TLR9 (rs5743836)	AA	114 (74.0)	36 (72.0)	0.819
	AG	34 (22.1)	11 (22.0)	
	GG	6 (3.9)	3 (6.0)	
TLR9 (rs352139)	TT	45 (29.2)	15 (30.0)	0.454
	TC	72 (46.8)	19 (38.0)	
	CC	37 (24.0)	16 (32.0)	
CD209 (rs735240)	GG	41 (26.6)	13 (26.0)	0.201
	GA	74 (48.1)	30 (60.0)	
	AA	39 (25.3)	7 (14.0)	
CD209 (rs4804803)	AA	85 (55.2)	35 (70.0)	0.115
	AG	62 (40.3)	12 (24.0)	
	GG	7 (4.5)	3 (6.0)	
IFNL3 (rs12979860)	CC	76 (49.4)	24 (48.0)	0.806
	CT	55 (35.7)	20 (40.0)	
	TT	23 (14.9)	6 (12.0)	
IFNL3 (rs8099917)	TT	117 (76.0)	34 (68.0)	0.197
	TG	33 (21.4)	12 (24.0)	
	GG	4 (2.6)	4 (8.0)	
TNF (rs1800629)	GG	124 (80.5)	35 (70.0)	0.297
	GA	28 (18.2)	14 (28.0)	
	AA	2 (1.3)	1 (2.0)	
IL10 (rs1800872)	TT	19 (12.3)	0 (0.0)	**0.033**
	TG	74 (48.1)	27 (54.0)	
	GG	61 (39.6)	23 (46.0)	
IL10 (rs1878672)	GG	63 (40.9)	14 (28.0)	0.165
	GC	69 (44.8)	30 (60.0)	
	CC	22 (14.3)	6 (12.0)	

*BKPyV* BK polyomavirus; *CTLA-4* cytotoxic T-lymphocyte antigen 4; *IL* interleukin; *N/A* not applicable; *SNP* single-nucleotide polymorphism; *TLR* toll-like receptor; *TNF* tumor necrosis factor.

^a^Bold characters indicate significant P-values.

Next, we explored the effect of the minor alleles of each SNPs analyzed in both dominant (heterozygous and homozygous) and recessive (homozygous only) states. Although none of the comparisons reached the *P* value required for statistical significance after applying the Bonferroni correction for multiple testing (set at 0.003), we observed some clear differences. The presence of the G allele of *IL10* (rs1800872) SNP in a dominant model (TG/GG) was apparently associated to an increased risk of BKPyV viremia as compared to the TT genotype (nominal *P* value = 0.009). The T allele of *TLR3* (rs3775291) SNP in a recessive model (TT) also showed a clear trend to increase the risk of the outcome (nominal *P* value = 0.024). Conversely, there was a non-significant tendency towards a lower incidence of BKPyV viremia when the G allele of the *CD209* (rs4804803) SNP was either in heterozygous or homozygous forms (AG/GG) (nominal *P* value = 0.065), suggesting a dominant protective role (Table 4).

**Table 4:** Associations between the cumulative incidence of BKPyV viremia and genotype combinatons of candidate SNPs according to dominant or recessive models.

Gene (SNP database ID number)	Model	Genotype	BKPyV viremia, n (%)		*P* value^a^
			No (N = 154)	Yes (N = 50)	
CTLA4 (rs5742909)	Dominant	CC	127 (82.5)	40 (80.0)	0.694
		CT/TT	27 (17.5)	10 (20.0)	
	Recessive	CC/CT	151 (98.1)	48 (96.0)	0.415
		TT	3 (1.9)	2 (4.0)	
CTLA4 (rs231775)	Dominant	AA	77 (50.0)	27 (54.0)	0.937
		AG/GG	77 (50.0)	23 (46.0)	
	Recessive	AA/AG	138 (89.6)	45 (90.0)	0.623
		GG	16 (10.4)	5 (10.0)	
TLR2 (rs5743708)	Dominant	GG	153 (99.4)	50 (100.0)	0.568
		GA/AA	1 (0.6)	0 (0.0)	
	Recessive	GG/GA	NA	NA	NA
		AA	NA	NA	
TLR3 (rs3775291)	Dominant	CC	79 (51.3)	22 (44.0)	0.370
		CT/TT	75 (48.7)	28 (56.0)	
	Recessive	CC/CT	139 (90.3)	39 (78.0)	**0.024**
		TT	15 (9.7)	11 (22.0)	
TLR9 (rs5743836)	Dominant	AA	114 (74.0)	36 (72.0)	0.778
		AG/GG	40 (26.0)	14 (28.0)	
	Recessive	AA/AG	148 (96.1)	47 (94.0)	0.529
		GG	6 (3.9)	3 (6.0)	
TLR9 (rs352139)	Dominant	TT	45 (29.2)	15 (30.0)	0.916
		TC/CC	109 (70.8)	35 (70.0)	
	Recessive	TT/TC	117 (76.0)	34 (68.0)	0.264
		CC	37 (24.0)	16 (32.0)	
CD209 (rs735240)	Dominant	GG	41 (26.6)	13 (26.0)	0.931
		GA/AA	113 (73.4)	37 (74.0)	
	Recessive	GG/GA	115 (74.7)	43 (86.0)	0.096
		AA	39 (25.3)	7 (14.0)	
CD209 (rs4804803)	Dominant	AA	85 (55.2)	35 (70.0)	0.065
		AG/GG	69 (44.8)	15 (30.0)	
	Recessive	AA/AG	147 (95.5)	47 (94.0)	0.679
		GG	7 (4.5)	3 (6.0)	
IFNL3 (rs12979860)	Dominant	CC	76 (49.4)	24 (48.0)	0.868
		CT/TT	78 (50.6)	26 (52.0)	
	Recessive	CC/CT	131 (85.1)	44 (88.0)	0.606
		TT	23 (14.9)	6 (12.0)	
IFNL3 (rs8099917)	Dominant	TT	117 (76.0)	34 (68.0)	0.264
		TG/GG	37 (24.0)	16 (32.0)	
	Recessive	TT/TG	150 (97.4)	46 (92.0)	0.087
		GG	4 (2.6)	4 (8.0)	
TNF (rs1800629)	Dominant	GG	124 (80.5)	35 (70.0)	0.119
		GA/AA	30 (19.5)	15 (30.0)	
	Recessive	GG/GA	152 (98.7)	49 (98.0)	0.720
		AA	2 (1.3)	1 (2.0)	
IL10 (rs1800872)	Dominant	TT	19 (12.3)	0 (0.0)	**0.009**
		TG/GG	135 (87.7)	50 (100.0)	
	Recessive	TT/TG	93 (60.4)	27 (54.0)	0.425
		GG	61 (39.6)	23 (46.0)	
IL10 (rs1878672)	Dominant	GG	63 (40.9)	14 (28.0)	0.102
		GC/CC	91 (59.1)	36 (72.0)	
	Recessive	GG/GC	132 (85.7)	44 (88.0)	0.683
		CC	22 (14.3)	6 (12.0)	

*BKPyV* BK polyomavirus; *CTLA-4* cytotoxic T-lymphocyte antigen 4; *IL* interleukin; *N/A* not applicable; *SNP* single-nucleotide polymorphism; *TLR* toll-like receptor; *TNF* tumor necrosis factor.

^a^Bold characters indicate significant *P* values.

### Impact of selected SNPs on BKPyV viremia-free survival

**Figure 1:**
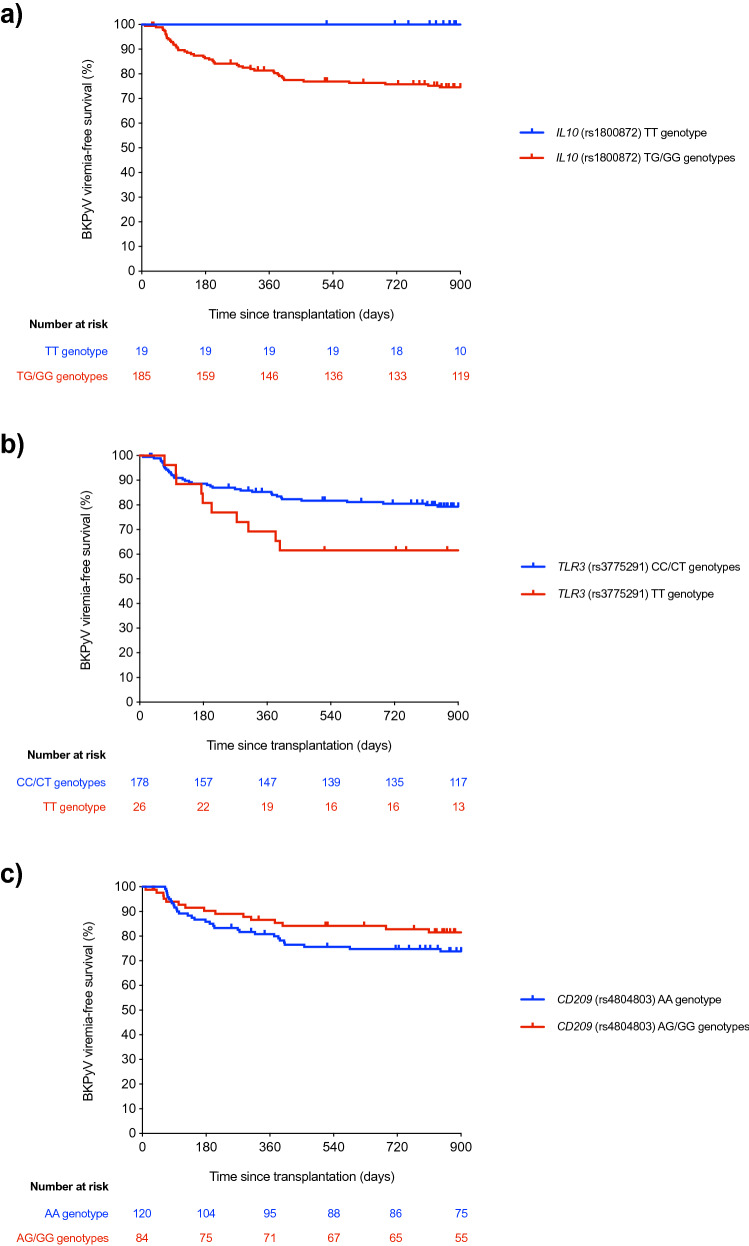
Comparison of Kaplan–Meier BKPyV viremia-free survival curves according to the genotype of selected SNPs (**a**) carriers of the TT genotype of *IL10* (rs1800872) (blue line) were compared to patients with TG/GG genotypes (red line) (log-rank *P* value = 0.015); (**b**) carriers of CC/CT genotypes of *TLR3* (rs3775291) (blue line) were compared to TT genotype carriers (red line) (log-rank *P* value = 0.026); and (**c**) carriers of the AA genotype of *CD209* (rs4804803) (blue line) were compared to those with AG/GG genotypes (red line) (log-rank *P* value = 0.096). BKPyV: BK polyomavirus; SNP: single-nucleotide polymorphism.

In view of the apparent impact observed for polymorphisms in the *IL10*, *TLR3* and *CD209* genes on the incidence of the study outcome, we compared BKPyV viremia-free survival curves between carriers and non-carriers of selected genotype combinations. Patients harboring the G allele of the *IL10* (rs1800872) SNP in heterozygous or homozygous states (TG/GG) were less likely to remain free from BKPyV viremia compared to TT genotype carriers (2-year survival rates: 73.5% vs. 100.0%, respectively; log-rank test *P* value = 0.015) (Fig. 1a). On the other hand, recipients bearing the T allele of the *TLR3* (rs3775291) SNP in the homozygous state (TT genotype) also exhibited lower BKPyV viremia-free survival in comparison to CC/CT carriers (2-year survival rates: 56.4% vs. 78.9%, respectively; log-rank test *P* value = 0.026) (Fig. 1b). Additionally, the dominant protective role observed for the *CD209* (rs4804803) SNP was also examined. Carriers of the G allele in heterozygous or homozygous forms (AG/GG) showed an increased BKPyV viremia-free survival than homozygotes for the reference A allele, although the difference did not achieve statistical significance (81.2% vs. 72.3%; log-rank test *P* value = 0.096) (Fig. 1c).

Subsequently we tested the impact of these SNPs by multivariable regression. To this end, Cox models were adjusted for those clinical variables found to be associated with the occurrence of BKPyV viremia by univariable analysis (Table S2). After controlling by recipient age, pre-transplant coronary heart disease, pre-transplant renal replacement therapy and donation after circulatory death (CDC), there was a significant association between the presence of the minor T allele of *TLR3* (rs3775291) SNP in homozygosis (TT genotype) and the occurrence of BKPyV viremia (adjusted hazard ratio [HR]: 2.16; 95% confidence interval [CI]: 1.08–4.30; *P* value = 0.029). In the case of *IL10* (rs1800872) SNP, the corresponding HR could not be calculated since no BKPyV events occurred among recipients bearing the reference allele in homozygosis (TT carriers). Finally, the protective effect linked to the G allele of the *CD209* (rs4804803) SNP (AG/GG genotypes) was also confirmed (adjusted HR: 0.54; 95% CI: 0.29–1.00; *P* value = 0.050) (models #1 in Table 5). To confirm the independent association between SNPs and study outcome, we further adjust the models for other clinically relevant variables despite the lack of univariate significance (acute graft rejection modelled as a time-dependent covariate and eGFR and tacrolimus trough levels at month 3). The HRs and corresponding 95% CIs remained essentially unchanged (models #2 in Table 5).

**Table 5:** Univariable and multivariable Cox regression models assessing the impact of selected SNPs on the incidence of BKPyV viremia during the post-transplant follow-up period.

	Univariable models			Multivariable models #1^a^			Multivariable models #2^b^		
	HR	95% CI	*P* value^c^	aHR	95% CI	*P *value^c^	aHR	95% CI	*P* value^c^
TG/GG genotype of *IL10* (rs1800872) SNP (versus TT)	–^d^	–	–	–^d^	–	–	–^d^	–	–
TT genotype of *TLR3* (rs3775291) SNP (versus CC/CT)	2.08	1.06–4.07	**0.032**	2.16	1.08–4.30	**0.029**	2.37	1.20–4.67	**0.013**
AG/GG genotype of *CD209* (rs4804803) SNP (versus AA)	0.59	0.32–1.09	0.092	0.54	0.29–1.00	**0.050**	0.55	0.29–1.00	0.052

*aHR* adjusted hazard ratio; *CI* confidence interval; *IL* interleukin; *SNP* single-nucleotide polymorphism; *TLR* toll-like receptor.

^a^Model #1 adjusted for recipient age, pre-transplant coronary heart disease, pre-transplant renal replacement therapy and donation after circulatory death (Table S1).

^b^Model #2 adjusted for recipient age, pre-transplant coronary heart disease, pre-transplant renal replacement therapy, donation after circulatory death, acute graft rejection (time-dependent covariate), estimated glomerular filtration rate and tacrolimus trough levels by month 3.

^c^Bold characters indicate significant *P* values.

^d^HRs were not estimable since all the cases of BKPyV infection occurred in recipient bearing TG/GG genotypes.

All these associations were essentially unchanged in a sensitivity analysis restricted to KT recipients of Caucasian ethnicity (Table S3). The low number of patients from other ethnic backgrounds hampered further population stratification.

### Additive effect of risk genotypes

**Figure 2:**
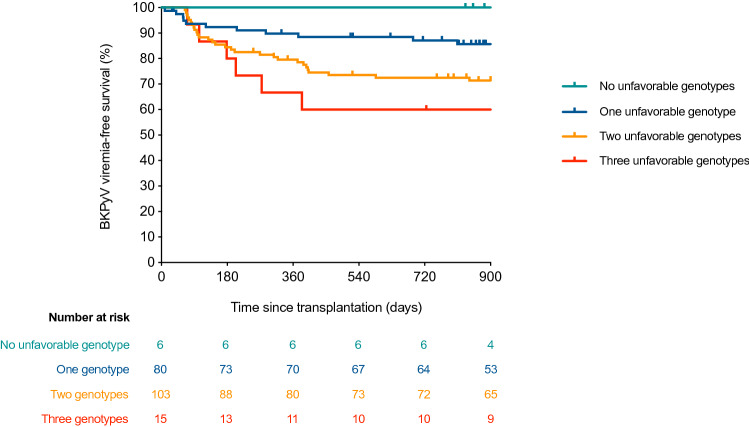
Comparison of Kaplan–Meier BKPyV viremia-free survival curves according to the number of unfavorable genotypes in selected SNPs: 0 (green line), 1 (blue line), 2 (orange line) and 3 unfavorable genotypes (red line) (log-rank *P* value = 0.008). Unfavorable genotypes were categorized as follow: TG/GG for *IL10* (rs1800872), TT for *TLR3* (rs3775291), and AA for *CD209* (rs4804803). BKPyV: BK polyomavirus.

We explored the potentially additive effect of the number of risk genotypes on the incidence of BKPyV viremia. In view of the protective role of the alternative G allele of *CD209* (rs4804803) SNP, we considered as “unfavorable” the presence of the reference allele in homozygous state (AA genotype). Patients were divided in different haplotypes according to the number of unfavorable SNP genotypes as follows: no unfavorable genotypes (6 patients [2.9%]), one genotype (80 [39.2%]), two genotypes (103 [50.5%]), and three genotypes (15 [7.4%]). We observed that the BKPyV viremia-free survival progressively decreased with the increasing number of unfavorable genotypes (2-year survival rates: 100.0% [no unfavorable genotypes], 85.4% [one genotype], 70.9% [two genotypes] and 52.5% [three genotypes]; log-rank test *P* value = 0.008) (Fig. 2). After adjusting for clinical variables, the number of unfavorable genotypes remained significant as a risk factor for BKPyV viremia (adjusted HR [per additional genotype]: 2.46; 95% CI: 1.53–3.94; *P* value < 0.001). As compared to the “clinical risk model”—which included recipient age, pre-transplant coronary heart disease, pre-transplant renal replacement therapy and DCD (Table S2)—, the addition of SNP genotyping information (as the number of unfavorable genotypes) increased the discriminative capacity of the “clinical-genetic risk model”, with area under the receiver operating characteristic curve (auROC) values of 0.692 (95% CI: 0.607–0.778) and 0.754 (95% CI: 0.678–0.830) for each model, respectively.

Finally, to test the hypothesis that the impact of SNPs in genes orchestrating innate responses would be more evident in the subgroup treated with agents that abrogate T-cell-mediated adaptive responses, we performed a sensitivity analysis restricted to patients that received antithymocyte globulin as induction therapy (n = 94). As compared to the overall cohort, the “clinical-genetic risk model” performed slightly better in this subgroup, with an auROC of 0.768 (955 CI: 0.658–0.879).

## Discusion

In this study we aimed to analyze the potential correlation between genetic polymorphisms located in genes coding for molecules mainly involved in innate immunity and the occurrence of BKPyV viremia after KT, as well as to evaluate whether the integration of SNP genotyping data with clinical variables might contribute to refine risk stratification. The results obtained are innovative since not much information is available on the genetic factors modulating the risk of BKPyV infection in the transplant setting^29,30^.

One novel finding is the correlation observed between the *IL10* (rs1800872) SNP and BKPyV viremia. The presence of the alternative G allele either in heterozygous or homozygous states conferred an increased risk of BKPyV viremia. In fact, no BKPyV events were observed among homozygous KT recipients for the reference allele (TT genotype). IL-10 is a key regulatory component of the immune response that suppresses T-cell proliferation, antigen-presenting cell functions and production of pro-inflammatory cytokines during the recovery stage following infection, in order to limit the inflammation-mediated tissue damage^34^. Previous studies have reported the effect of *IL10* polymorphisms on the susceptibility to human immunodeficiency virus infection^35,36^ or the risk of HBV-induced liver damage and progression to chronicity^37^. A large proportion of the interindividual variability observed in the production of human IL-10 is attributable to genetic variation in the highly polymorphic promoter region of the *IL10* gene, where the rs1800872 SNP is located^38^. Sadeghi et al. showed that BKPyV-positive KT recipients, especially those with high viral loads, had stronger inflammatory cytokine responses in urine than BKPyV-negative patients^39^. Therefore, it could be hypothesized that haplotypes associated to decreased IL-10 expression would favor BKPyV replication by inducing a pro-inflammatory milieu, although further research is needed to clarify the underlying mechanism.

We have also shown that homozygous carriers of the minor T allele (TT genotype) of the *TLR3* (rs3775291) SNP had a two-fold increased risk of BKPyV viremia, even after controlling for clinical factors—such as recipient age or type of donor—by multivariable regression. TLR3 is an internal PRR localized in the endosomal vesicle that senses dsRNA^1,18^, which is produced as an intermediate product of genomic RNA replication in cells infected with positive-sense RNA viruses^40–42^. The sensing cascade is activated upon recognition of dsRNA by Toll/IL-1 receptor-domain containing adapter-inducing IFN-β (TRIF)^43^. Once recruited to the intracellular domain of TLR3, the TRIF-mediated pathway induces the activation of nuclear factor κB (NF-κB) and IFN regulatory factor 3 (IFR3), which migrate to the nucleus and stimulate the production of type I IFNs^44^. Therefore, TLR3 plays a pivotal role in host defense against viral infection^45^.

The rs3775291 SNP is a missense mutation in the *TLR3* gene (C to T transition at nucleotide 1234) that replaces a conserved leucine with phenylalanine (L412F). This substitution does not affect the expression of TLR3 but impairs its dimerization at the membrane and decreases its binding capacity to dsRNA, resulting in a lower signaling activity compared to the wild-type form^46^. This detrimental effect on the sensing function of TLR3 is supported by multiple studies that correlate this SNP with the susceptibility to viral infections, such as CMV disease, hepatitis B and C, TBE or chikungunya^47–51^. It has been also associated with the occurrence of herpes simplex encephalitis in the general population^52,53^ and microcephaly in newborns from pregnant women infected with Zika virus^54^, highlighting the importance of the TLR3/TRIF pathway in the central nervous system.

The role of TLR3 and TRIF in the innate response against BKPyV has been shown in an in vitro model using polyriboinosinic:polyribocytidylic acid (poly[I:C]) as a dsRNA analog to trigger the sensing cascade^18^. Ribeiro et al. found that TLR3 expression in collecting duct epithelial cells was enhanced upon stimulation with poly(I:C) and proinflammatory cytokines. In addition, activation of TLR3 and RIG-I by poly(I:C) induced the expression of cytokines, chemokines and IFN-β mRNA, and this inflammatory response could be blocked by small interfering RNA to TLR3. These results collectively suggest that the activation of innate immune mechanisms via TLR3 is involved in the antiviral and inflammatory responses to BKPyV, and that a deregulated signaling may contribute to the pathogenesis of BKPyVAN^18^. These authors did not investigate the role of genetic diversity within the *TLR3* gene. Nevertheless, if TLR3 is an important actor during BKPyV infection, it is plausible that homozygote carriers of a deleterious mutation that leads to impaired function would be less able to mount an effective immune response, as supported by our data.

Finally, an intriguing finding is the apparent beneficial effect provided by the minor G allele of *CD209* (rs4804803) SNP, both in heterozygous and homozygous states. The gen *CD209* codes for DC-SIGN, a transmembrane PRR belonging to the CLR family. DC-SIGN mediates cell-to-cell adhesion acting as a high affinity receptor and plays a relevant role in T-cell activation^55^. In addition, DC-SIGN has been shown to facilitate cell binding and entry for many viruses, such as CMV, Ebola virus, Japanese encephalitis virus, influenza virus or severe acute respiratory syndrome coronavirus 2 (SARS-CoV-2)^56,57^. Of note, the presence of the G allele has been also associated with a lower susceptibility to tuberculosis disease and severe dengue^58,59^, which is consistent with the results presented herein. The rs4804803 SNP is located at the 5’UTR region of the promoter region of *CD209* and results in an adenine to guanine substitution at position -336 (-336A/G). The presence of the minor G allele decreases the transcription level of *CD209* and downregulates DC-SIGN expression in DCs^58–60^. It has been reported that low pre-transplant numbers of peripheral blood DCs increases the risk of BKPyV viremia after KT^26^, and that DCs levels are decreased in patients with BKPyVAN as compared to those with normal graft function^11^. These results point to a role for DCs in the response against BKPyV. The expected decrease in *CD209* expression among G allele carriers might result in a lower susceptibility of DCs to BKPyV, although the biological rationale for this protective effect remains unclear.

Our study has some limitations to be acknowledged, the most important of which is the absence of cases of biopsy-proven BKPyVAN. Indeed, the small sample size limited the number of BKPyV events, weakening the statistical power. In this regard, we considered BKPyV viremia at any level as study outcome in view of the low number of patients in our cohort that developed DNAemia > 4.0 log_10_ copies/mL or presumptive BKPyVAN (8 and 3 cases, respectively). It should be noted, however, that we observed an increasing trend in the incidence of high-level viremia according to the number of unfavorable genotypes (0.0%, 2.5%, 4.9% and 6.7% for none, one, two and three genotypes, respectively). Only a few patients with high-level BKPyV DNAemia underwent renal biopsy, although it should be noted that the clinical guidelines developed by the American Society of Transplantation Infectious Diseases Community of Practice (AST‐IDCOP) states that histological examination may be dispensable in KT recipients with baseline renal function and standard immunological risk, in which the diagnosis of “presumptive” BKPyVAN may be assumed and the immunosuppression accordingly tappered^16^. The presence of any-level BKPyV viremia acts as a surrogate for the subsequent development of high-level viremia and organ-invasive disease within a continuous spectrum, with historically high rates of progression to BKPyVAN among BKPyV-DNAemic patients^16,61^. On the other hand, it was not possible to analyze the effect of the rare A allele of *TLR2* (rs5743708) SNP since 99.5% of patients were homozygotes for the reference G allele. Finally, we did not perform a complete haplotype analysis for the studied genes.

In conclusion, the present study contributes to a better understanding of the host genetic factors that modulate the risk of BKPyV infection among KT recipients and demonstrate for the first time the impact on this event of polymorphisms within *TLR3, IL10* and *CD209* genes. In addition, from a clinical perspective it is noteworthy that half of the patients exhibited at least two unfavorable genotypes. Thus, the integration of selected SNP genotyping data with clinical variables might result in a meaningful improvement in the ability to predict the occurrence of post-transplant BKPyV viremia. Any mechanistic explanation on how the presence of a given allele in the selected SNPs modulates the risk of BKPyV viremia should be considered as merely tentative. Further studies should confirm the association of these SNPs with the incidence of BKPyVAN and eventually elucidate the underlying biological and functional mechanisms.

## Methods

### Study population and setting

The present study was based on a prospectively maintained database that included all consecutive adult patients undergoing KT at our institution between November 2014 and December 2016. Double organ recipients (e.g. kidney-pancreas) and patients experiencing graft loss within the first post-transplant week were excluded. The study was performed in accordance with the ethical standards laid down in the Declarations of Helsinki and Istanbul. All the patients gave their informed consent and the local Clinical Research Ethics Committee of the Hospital 12 de Octubre approved the study protocol (number 14/030). The paper was prepared in accordance with the methodological recommendations drawn by the STREGA initiative.

### Study design

The study outcome was the occurrence of BKPyV viremia at any level during the post-transplant period. Participants were enrolled at the time of KT and followed-up for at least 12 months, unless graft loss (retransplantation or permanent return to dialysis) or death occurred earlier. Scheduled follow-up visits were carried out at baseline, every 2 weeks during the first 3 months, and monthly thereafter, as well as whenever clinically indicated. Pre-transplant, peri-operative and post-transplant variables were prospectively recorded by means of a standardized case report form, and pseudo-anonymized data were entered into a secure REDCap database. Descriptions of immunosuppression and prophylaxis regimens are detailed in Supplementary Methods.

### Management of BKPyV infection

Maintenance immunosuppression was reduced according to the criteria of the attending nephrologist in those KT recipients in which BKPyV viremia was documented. To this end, tacrolimus trough levels were commonly targeted to < 6 ng/mL and/or daily doses of mycophenolate mofetil or enteric-coated mycophenolate sodium were halved or discontinued. Conversion to low-dose mammalian target of rapamycin (mTOR) inhibitor and tacrolimus (target trough levels of 4–6 ng/mL) was performed in selected cases with sustained BKPyV viremia. Other approaches, such as switching from tacrolimus to low-dose cyclosporine or use of agents with potential anti-BKPyV activity (i.e. leflunomide or quinolones), were not systematically applied. The indication to perform a renal biopsy was restricted to the presence of increasing BKPyV DNAemia (> 4.0 log_10_ copies/mL in more than one point) despite immunosuppression tapering in association with a significant decrease of graft function in a patient at high immunological risk, in line with the most recent AST‐IDCOP guidelines^16^. In the absence of the two later criteria, the diagnosis of “presumptive” BKPyVAN (as defined below) was assumed by the nephrologist in charge.

### Study definitions

BKPyV viremia was defined by the detection of plasma BKPyV DNAemia at any level by real-time polymerase chain reaction (PCR), as detailed below. The presence of viruria or decoy cells in urine cytology specimens was not investigated. The diagnosis of BKPyVAN was categorized as “proven” (demonstration of cytopathic changes in tubular epithelial cells, later confirmed by means of immunohistochemistry or in situ hybridization) or “presumptive” (plasma BKPyV DNAemia > 4.0 log_10_ copies/mL with an increase in one of two measurements performed within 3 weeks or less) according to the AST‐IDCOP guidelines^16,17^. Other study definitions are provided in Supplementary Methods.

### Monitoring of BKPyV viremia

Plasma BKPyV viral load was assessed by a commercial real-time PCR assay (RealStar^®^ BKV PCR Kit 1.0, Altona Diagnostics GmbH, Hamburg, Germany). DNA was extracted from 200 μL of sample with the NucliSENS^®^ easyMag^®^ instrument (bioMérieux Diagnostics, Marcy l’Etoile, France), according to the manufacturer's instructions. BKPyV viremia was assessed per protocol fortnightly during the first two post-transplant months and on a monthly basis thereafter during the first post-transplant year. In addition, quantification of BKPyV DNAemia was further ordered by the attending physician beyond this schedule when BKPyVAN was suspected. Viral loads were log_10_-transformed for analysis. According to the manufacturer, the analytical sensitivity of the RealStar^®^ BKV PCR Kit 1.0 is 0.712 copies/μL (95% CI: 0.404–1.693).

### SNP genotyping

Whole blood specimens that have been collected at patient inclusion and stored at − 80 °C were retrieved for the analyses described herein. DNA was extracted with the KingFisher™ Duo Prime (ThermoFisher Scientific Inc, Waltham, MA) using the MagMax DNA Multi-Sample Ultra 2.0 kit, following the manufacturer´s instructions. *TLR2* (rs5743708), *TLR3* (rs3775291), *TLR9* (rs5743836, rs352139), *CD209* (rs735240, rs4804803), *IFNL3* (rs12979860, rs8099917), *TNF* (rs1800629), *IL10* (rs1878672, rs1800872), and *CTLA4* (rs5742909, rs231775) genotyping was performed by TaqMan technology (ThermoFisher Scientific) in a QuantStudio 3 system (Applied Biosystems, Foster City, CA). SNP and allele (genotype) calling was made by a standard end-point analysis with the aid of a commercial genotype-calling software (TaqMan™ Genotyper Software v1.0.1) and the QuantStudio Design and Analysis Software v1.5.1 (both from Applied Biosystems).

### Statistical analysis

Quantitative data were shown as the mean ± standard deviation or the median with IQR. Qualitative variables were expressed as absolute and relative frequencies. Within-patient differences in eGFR across different time points were compared with the T-student test for repeated measures. Normality of the distributions was tested with the Kolgomorov-Smirnov test. Deviation from the Hardy–Weinberg equilibrium for each SNP was evaluated by the *χ*^2^ test with one degree of freedom. Comparisons of the cumulative incidence of BKPyV infection between recipients with different genotypes for the SNPs investigated were performed by the *χ*^2^ test or the Fisher’s exact test, as appropriate. Additional pairwise comparisons were conducted between different SNP genotype groups, either individually or in combination. Survival probabilities were estimated by the Kaplan–Meier method with BKPyV infection as event, and differences between groups were compared by the log-rank test. Multivariable Cox regression models (enter method) were constructed to investigate the association between selected SNPs and BKPyV viremia, with results expressed as HRs and 95% CIs. Only those variables showing *P* values ≤ 0.05 at the univariate level were entered into the model. The most parsimonious model (i.e. the highest outcome variability explained with the lowest number of variables) was selected for the construction of a model based on clinical variables only (“clinical risk model”). We also performed a haplotype analysis by creating a score based on those SNPs found to exert an independent impact on the outcome (a point was added for each unfavorable genotype carried by the patient). We then compared the discriminative accuracy, quantified by the auROC, of the “clinical risk model” with that of the model also incorporating SNP genotyping ("clinical-genetic risk model”). All the significance tests were two-tailed and considered as significant at a *P* value < 0.05. The Bonferroni correction method (the α value for each comparison equal to the fixed α value divided by the total number of comparisons) was applied to avoid the possible inflation of *P*-values owing to multiple comparisons. Statistical analysis was performed using SPSS v21 (Statistical Package for Social Sciences, Chicago, IL) and graphs were generated with Prism v6.0 (GraphPad Software Inc., La Jolla, CA).

## Supplementary Information

Supplementary Information.

## Data Availability

The data that support the findings of this study are available upon reasonable request to the corresponding author.
