# Supplementary material for: Genetic polymorphisms in TLR3, IL10 and CD209 influence the risk of BK polyomavirus infection after kidney transplantation
Source: Sci Rep. 2022 Jul 5;12:11338. doi: 10.1038/s41598-022-15406-0 (PMC9255529; doi:10.1038/s41598-022-15406-0)
Supplement: Supplementary file 1 — Supplementary Information. [file 41598_2022_15406_MOESM1_ESM.pdf]

Genetic polymorphisms in *TLR3*, *IL10* and *CD209* influence the risk of BK polyomavirus infection after kidney transplantation.

Natalia Redondo[1,2,*], Isabel Rodríguez-Goncer[1,2], Patricia Parra[1,2], Francisco López-Medrano[1,2,3], Esther González[4], Ana Hernández[4], Hernando Trujillo[4], Tamara Ruiz-Merlo[1,2], Rafael San Juan[1,2,3], María Dolores Folgueira[3,5], Amado Andrés[3,4], José María Aguado[1,2,3], Mario Fernández-Ruiz[1,2,3]

1. Unit of Infectious Diseases, Hospital Universitario "12 de Octubre", Instituto de Investigación Sanitaria Hospital "12 de Octubre" (imas12), Madrid, Spain.
2. Centro de Investigación Biomédica en Red (CIBER) en Enfermedades Infecciosas, Spain.
3. Department of Medicine, School of Medicine, Universidad Complutense, Madrid, Spain.
4. Department of Nephrology, Hospital Universitario "12 de Octubre", Instituto de Investigación Sanitaria Hospital "12 de Octubre" (imas12), Madrid, Spain.
5. Department of Microbiology, Hospital Universitario "12 de Octubre", Instituto de Investigación Sanitaria Hospital "12 de Octubre" (imas12), Madrid, Spain.

**** Corresponding author:*** Natalia Redondo, BSc, PhD. Unit of Infectious Diseases. Hospital Universitario "12 de Octubre". Centro de Actividades Ambulatorias, 6ª planta, bloque D. Avda. de Córdoba, s/n. Postal code 28041. Madrid, Spain. Phone: +34 913908000. Fax: +34 914695775. E-mail address: natalia.redondo.imas12@h12o.es

<u>Supporting Material</u>

Supplementary Methods

Description of immunosuppression and prophylaxis regimens

Induction therapy with intravenous (IV) rabbit antithymocyte globulin (ATG-Fresenius®, 1.25 mg/Kg daily for 5-7 days) was used in the case of donation after circulatory death, with delayed introduction of the calcineurin inhibitor (CNI) from post-transplant day 6. Recipients at high immunological risk (peak panel-reactive antibody >50%, second transplantation in case the first graft was lost to rejection within two years, or third or fourth graft) also received ATG induction for 1-3 days with early CNI initiation from post-transplant day 0. Basiliximab induction (20 mg on days 0 and 4) with delayed CNI introduction on day 5 was reserved to patients at increased risk for CNI-related nephrotoxicity (i.e., older age or pre-transplant comorbidities). Standard maintenance immunosuppression consisted of tacrolimus (0.1 mg/Kg daily, adjusted to a target trough level of 10-15 ng/mL during the first month and 5-10 ng/mL thereafter); mycophenolate mofetil (1,000 mg twice daily) or enteric-coated mycophenolic acid (360 mg twice daily); and prednisone (1 mg/Kg daily with progressive tapering). Conversion to a mammalian target of rapamycin inhibitor-based regimen with reduced-dose tacrolimus (target trough levels of 3-6 ng/mL) was performed on an individual basis for recipients experiencing severe CNI-related adverse effects, difficult-to-treat cytomegalovirus (CMV) infection, sustained BK polyomavirus viremia or post-transplant *de novo* malignancy.

All patients received as preoperative antibiotic prophylaxis a single IV dose of cefazolin (which was replaced with ciprofloxacin in the case of hypersensitivity to ß-lactams). Prophylaxis against *Pneumocystis jirovecii* pneumonia was based on trimethoprim-sulfamethoxazole (160/800 mg three times weekly) or monthly aerosolized pentamidine (300 mg) for 9 months. In patients at high-risk for CMV infection, universal prophylaxis with oral valganciclovir (VGC) (900 mg daily) was given for 3 months (seropositive recipients [R+] receiving induction therapy with ATG) or 6 months (serology mismatch [D+/R-]). Intermediate-risk patients (R+ without ATG induction)

were managed by polymerase chain reaction (PCR)-guided pre-emptive therapy, and IV GCV (5 mg/Kg/12 hours) or oral VGCV (900 mg/12 hours) for at least 2 weeks was initiated in the presence of high level or rapidly increasing CMV viral load. (V)GCV doses were adjusted according to renal function when necessary.

Additional study definitions

CMV infection was defined by the demonstration of CMV DNAemia by real-time PCR regardless of the presence of attributable symptoms or other clinical manifestations. Viral syndrome was defined by the presence of CMV infection plus fever plus at least one of the following criteria: leukopenia (white blood cell [WBC] count <3.50 x 10^3 cells/µL if baseline WBC count was ≥4.00 x 10^3 cells/µL or a decrease >20% if baseline WBC count was <4.00 x 10^3 cells/µL); atypical lymphocytosis (≥5%); thrombocytopenia (platelet count <100 x 10^3 cells/µL if baseline count was ≥115 x 10^3 cells/µL or a decrease >20% if baseline platelet count was <115 x 10^3 cells/µL); or elevation of ALT or AST of more than 2 times the upper limit of normal. End-organ CMV disease included either probable or proven categories, with the latter requiring the documentation of CMV replication in tissue specimens by viral culture, immunohistochemistry, histopathology, or DNA hybridization, in the presence of attributable clinical manifestations. The graft function was assessed by estimated glomerular filtration rate using the abbreviated Modification of Diet in Renal Disease (MDRD-4) equation. Delayed graft function denoted the requirement for dialysis within the first 2 weeks after transplantation. Acute graft rejection was suspected in case of sudden deterioration of graft function and diagnosed by histological examination. If renal biopsy was not technically possible, empirically treated episodes that responded to steroid boluses were also taken into account. Graft loss was defined by permanent return to dialysis, allograft nephrectomy, and/or the need of retransplantation.

Supplementary Results

Figure S1. Kaplan-Meier curve of the cumulative incidence of any-level BKPyV viremia during the study period. BKPyV: BK polyomavirus.

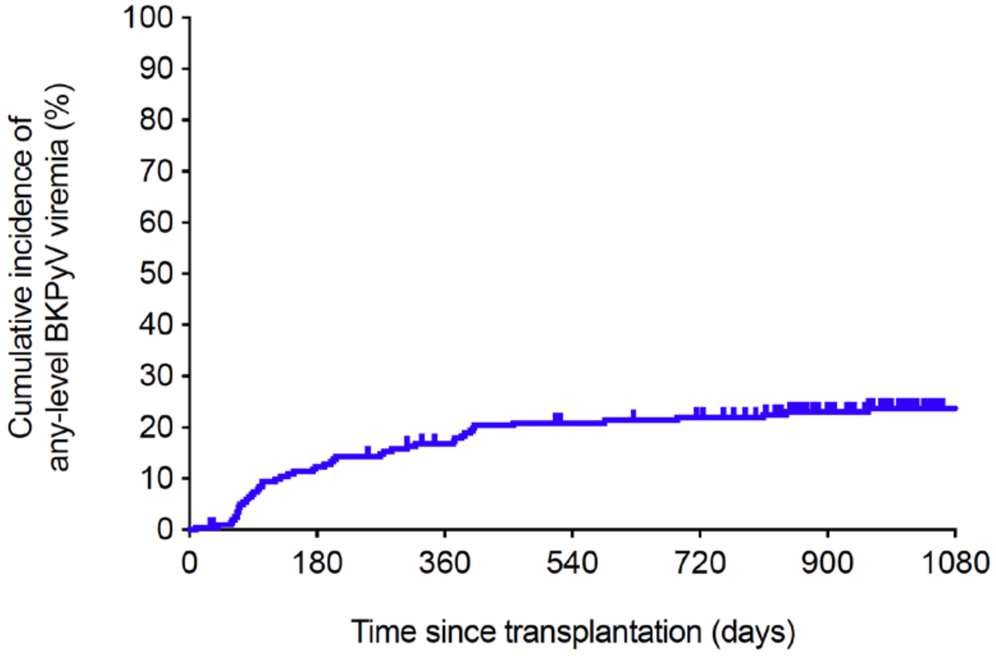

Number at risk	204	178	165	154	150	128	89

Table S1. Genotype frequencies and deviation from the Hardy-Weinberg equilibrium in candidate SNPs in the study cohort.

Gene (SNP database ID number)	Genotype	(%)	Genotypic frequencies		X^2
			Expected according to the HWE	Observed	
CTLA4 (rs5742909)	CC	81.9	164.2	167	4.63
	CT	15.7	37.7	32	
	TT	2.5	2.2	5	
CTLA4 (rs231775)	AA	51.0	100.9	104	1.05
	AG	38.7	85.1	79	
	GG	10.3	17.9	21	
TLR2 (rs5743708)	GG	99.5	203.0	203	0.0012
	GA	0.5	0.9	1	
	AA	0.0	0.0	0	
TLR3 (rs3775291)	CC	49.5	95.4	101	3.30
	CT	37.7	88.2	77	
	TT	12.7	20.4	26	
TLR9 (rs5743836)	AA	73.5	145.9	150	4.92
	AG	22.1	53.3	45	
	GG	4.4	4.9	9	
TLR9 (rs352139)	TT	29.4	54.6	60	2.33
	TC	44.6	101.9	91	
	CC	26.0	47.6	53	
CD209 (rs735240)	GG	26.5	55.1	54	0.09
	GA	51.0	101.8	104	
	AA	22.5	47.1	46	
CD209 (rs4804803)	AA	58.8	120.8	120	0.11
	AG	36.3	72.3	74	
	GG	4.9	10.8	10	
IFNL3 (rs12979860)	CC	49.0	92.7	100	5.44
	CT	36.8	89.7	75	
	TT	14.2	21.7	29	
IFNL3 (rs8099917)	TT	74.0	147.6	151	3.59
	TG	22.1	51.9	45	
	GG	3.9	4.6	8	
TNF (rs1800629)	GG	77.9	158.8	159	0.01
	GA	20.6	42.4	42	
	AA	1.5	2.8	3	
IL10 (rs1800872)	TT	9.3	23.7	19	
	TG	49.5	91.6	101	2.13

	GG	41.2	88.7	84	
IL10 (rs1878672)	GG		78.4	77	0.18
	GC		96.1	99	
	CC		29.4	28	

CTLA-4: cytotoxic T-lymphocyte antigen 4; HWE: Hardy-Weinberg equilibrium; *TNF:* tumor necrosis factor; IL: interleukin; SNP: single-nucleotide polymorphism; TLR: toll-like receptor.

Table S2. Univariable analysis of clinical factors associated to the occurrence of BKPyV viremia (study outcome) during the follow-up period.

	**No BKPyV viremia** (N = 154)	**BKPyV viremia** (N = 50)	***P*-value**	**Univariate analysis**		
				HR	95% CI	*P*-value
Age of recipient, years [mean ± SD]	53,0 ± 15,4	59.6 ± 15.7	0.010	1.30[d]	1.07 – 1.57	0.008
Gender (male) [n (%)]	108 (70.1)	38 (76.0)	0.424			
BMI of recipient, Kg/m^2 [mean ± SD][a]	25.3 ± 10.7	25.7 ± 3.6	0.654			
Pre-transplant diabetes mellitus [n (%)]	42 (27.3)	16 (32.0)	0.520			
Pre-transplant coronary heart disease [n (%)]	34 (22.1)	22 (44.0)	0.002	2.46	1.40 – 4.31	0.002
Pre-transplant chronic lung disease [n (%)]	21 (13.6)	6 (12.0)	0.767			
Previous solid organ transplantation [n (%)]	20 (12.9)	8 (16.0)	0.591			
Pre-transplant renal replacement therapy [n (%)]	132 (85.7)	48 (96.0)	0.050	3.54	0.86 – 14.57	0.080
Time on dialysis, months [median (IQR)][b]	17.3 ± 51.7	14.9 ± 49.6	0.937			
Positive HCV serostatus [n (%)]	11 (7.4)	4 (8.0)	0.886			
Age of donor, years [mean ± SD]	53.2 ± 15.0	55.7 ± 18.2	0.388			
DCD donor [n (%)]	40 (25.9)	6 (12.0)	0.040	0.45	0.19 – 1.05	0.066
Living donor [n (%)]	25 (16.2)	4 (8.0)	0.147			
CMV serostatus D-/R+ [n (%)]	15 (9.7)	8 (16.0)	0.224			
Cold ischemia time, hours [mean ± SD][c]	16.1 ± 8.4	17.4 ± 6.8	0.268			
Number of HLA mismatches [median (IQR)]	4 (3 – 5)	4 (3 – 5)	0.814			
Induction therapy with ATG [n (%)]	76 (49.4)	18 (36.0)	0.100			
Induction therapy with basiliximab [n (%)]	61 (39.6)	22 (44.0)	0.583			
Delayed graft function [n (%)]	75 (48.7)	24 (48.0)	0.973			
eGFR at month 1, mL/min/1.72 m^2 [mean ± SD]	41.0 ± 18.5	39.6 ± 19.7	0.666			

eGFR at month 3, mL/min/1.72 m^2 [mean ± SD]	43.8 ± 16.3	41.4 ± 16.4	0.359
eGFR at month 6, mL/min/1.72 m^2 [mean ± SD]	44.6 ± 15.6	41.9 ± 16.2	0.305
Use of mTOR inhibitor [n (%)]			
During the first 180 post-transplant days	3 (1.9)	3 (6.0)	0.141
During the first 360 post-transplant days	9 (5.8)	7 (14.0)	0.062
Acute graft rejection [n (%)]			
During the first post-transplant month	6 (3.9)	1 (2.0)	0.522
During the first 3 post-transplant months	7 (4.5)	3 (6.0)	0.679
During the first 6 post-transplant months	14 (9.1)	5 (10.0)	0.848

ATG: antithymocyte globulin; BMI: body mass index; BKPyV: BK polyomavirus; CI: confidence interval; CMV: cytomegalovirus; D: donor; DCD: donation after circulatory death; eGFR: estimated glomerular filtration rate; HLA: human leukocyte antigen; HR: hazard ratio; IQR: interquartile range; mTOR: mammalian target of rapamycin; R: recipient; SD: standard deviation.

[a] Data on BMI was not available for 17 patients.

[b] Data on time on dialysis was not available for 24 patients.

[c] Data on cold ischemia time was not available for 5 patients.

[d] HR per each ten-year increment.

Table S3. *Sensitivity analysis:* Univariable and multivariable Cox regression models assessing the impact of selected SNPs on the incidence of BKPyV viremia during the post-transplant follow-up period in the subgroup of recipients from Caucasian ethnicity (n = 177).

	Univariable models			Multivariable models[a]		
	HR	95% CI	*P*-value	aHR	95% CI	*P*-value
TG/GG genotype of *IL10* (rs1800872) SNP (versus TT)	-[b]	-	-	-[b]	-	-
TT genotype of *TLR3* (rs3775291) SNP (versus CC/CT)	2.51	1.19 – 5.26	0.030	2.29	1.08 – 4.92	0.035
AG/GG genotype of *CD209* (rs4804803) SNP (versus AA)	0.57	0.29 – 1.11	0.099	0.53	0.27 – 1.02	0.058

aHR: adjusted hazard ratio; CI: confidence interval; IL: interleukin; SNP: single-nucleotide polymorphism; TLR: toll-like receptor.

[a] Model adjusted for recipient age, pre-transplant coronary heart disease, pre-transplant renal replacement therapy and donation after circulatory death (**Table S3**).

[b] HRs were not estimable since all the cases of BKPyV infection occurred in recipient bearing TG/GG genotypes.
